# Engineering and characterization of a novel Self Assembling Protein for *Toxoplasma* peptide vaccine in HLA-A*11:01, HLA-A*02:01 and HLA-B*07:02 transgenic mice

**DOI:** 10.1038/s41598-020-73210-0

**Published:** 2020-10-12

**Authors:** Kamal El Bissati, Ying Zhou, Sara M. Paulillo, Senthil K. Raman, Christopher P. Karch, Steve Reed, Ashley Estes, Amber Estes, Joseph Lykins, Peter Burkhard, Rima McLeod

**Affiliations:** 1 Institute of Molecular Engineering, The University of Chicago Medical Center, 5841 S. Maryland Ave, Chicago, IL 60637 USA; 2 Department of Ophthalmology and Visual Sciences, The University of Chicago, 5841 S. Maryland Ave, Chicago, IL 60637 USA; 3 Alpha-O Peptides AG, Lörracherstrasse 50, 4125 Riehen, Switzerland; 4 Institute of Materials Science and Department of Molecular and Cell Biology, University of Connecticut, 97 North Eagleville Road, Storrs, CT 06269 USA; 5 Infectious Diseases Research Institute, 1616 Eastlake Ave E, Suite 400, Seattle, WA 98102 USA; 6 Department of Pediatrics (Infectious Diseases), The University of Chicago, 5841 S. Maryland Ave, Chicago, IL 60637 USA

**Keywords:** Protein vaccines, Parasite host response

## Abstract

Fighting smart diseases requires smart vaccines. Novel ways to present protective immunogenic peptide epitopes to human immune systems are needed. Herein, we focus on Self Assembling Protein Nanoparticles (SAPNs) as scaffolds/platforms for vaccine delivery that produce strong immune responses against *Toxoplasma gondii* in HLA supermotif, transgenic mice. Herein, we present a useful platform to present peptides that elicit CD4^+^, CD8^+^ T and B cell immune responses in a core architecture, formed by flagellin, administered in combination with TLR4 ligand-emulsion (GLA-SE) adjuvant. We demonstrate protection of HLA-A*11:01, HLA-A*02:01, and HLA-B*07:02 mice against toxoplasmosis by (i) this novel chimeric polypeptide, containing epitopes that elicit CD8^+^ T cells*,* CD4^+^ T helper cells, and IgG2b antibodies, and (ii) adjuvant activation of innate immune TLR4 and TLR5 pathways. HLA-A*11:01, HLA-A*02:01, and HLA-B*07:02q11 transgenic mouse splenocytes with peptides demonstrated predicted genetic restrictions. This creates a new paradigm-shifting vaccine approach to prevent toxoplasmosis, extendable to other diseases.

## Introduction

**Figure 1:**
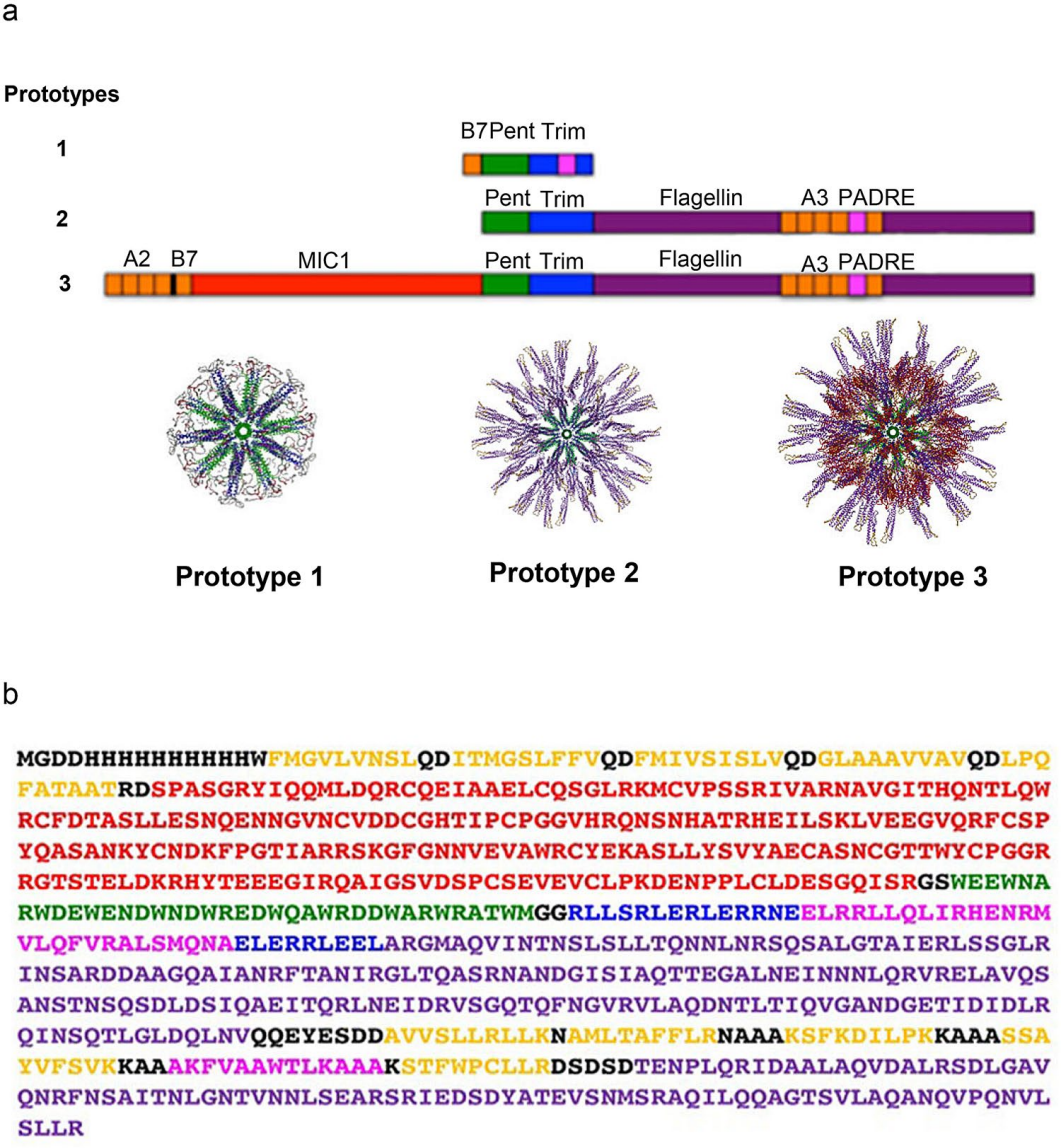
(**a**) Prototype constructs used for SAPN studies. The core particle of different SAPN constructs used for vaccine studies against toxoplasmosis was composed of the pentameric (green) and trimeric (blue) coiled coils. Attached to the core are the TLR5 agonist flagellin (purple) and the B cell epitope MIC1 (red) and depending on the particular construct the CD8^+^ epitopes (orange) and CD4+ epitopes (magenta) are either engineered into flagellin or the trimeric coiled coil or attached to the N-terminal end of the protein chain. Prototype 1 (P1)^12^ incorporates the T. gondii B07 epitope LPQFATAAT (GRA720–28) and an universal CD4^+^ T-cell epitope (derived from PADRE) into the SAPN. Prototype 2 (P2)^11^ incorporates five HLA-A*11:01-restricted CD8^+^ epitopes KSFKDILPK(SAG1_224-232_), STFWPCLLR(SAG2C_13-21_), AVVSLLRLLK(GRA5_89-98_), SSAYVFSVK(SRS52A_250-258_), and AMLTAFFLR(GRA6_164-172_), and the universal CD4^+^ T-cell epitope. All are integrated into a flagellin sequence as a component of the nanoparticle to make it a self-adjuvanted SAPN. Prototype 3 (P3) (design construct of the current study), in addition to the five HLA-A*11:01-restricted CD8^+^ epitopes and PADRE, the B cell MIC1 protein, 4 HLA-A*02:01-restricted CD8^+^ epitopes, and one HLA-B*07:01-restricted CD8^+^ epitope are attached to the N-terminal end of the protein chain. (**b**) Gene cloning of nanoparticle proteins. The polypeptide was cloned between the NdeI/EcoRI restriction sites in pET23b vector (Novagen) and yield the following sequence (see more details in the “Materials and methods”).

*Toxoplasma gondii* causes serious human illness. This apicomplexan, obligate, intracellular, parasite can infect all human cells, especially those of the brain and eye. Immunocompromised individuals, the fetus and newborn infant are most severely affected^1^. Severe active infection can cause death, respiratory failure as well as harm other organs, even in those without known immune compromise. Encephalitis and ophthalmologic disease cause substantial morbidity. Although anti-parasitic medicines, i.e., pyrimethamine and sulfadiazine are effective against tachyzoites, they do not eradicate dormant, encysted parasite forms^1,2^. Thus, one of the aspects of our study, is to address the gap of a substantial need to develop a potent and safe vaccine. This work builds on our foundation of novel work using a bioinformatics/immunosense/empiric approach with human cells and HLA transgenic mice^3–7^. We defined panels of octamer/nonamer peptides that bind to major HLA Class I supermotifs. IFN-γ producing CD8^+^ T cells specific to these peptides were detected in individuals with three Human Leukocyte Antigen (HLA) supermotifs (HLA supertype A03, A02, and B07) present in large proportions of the world population. We used peptides that bind to a subset of these HLA alleles to immunize HLA supermotif transgenic mice as a proof of principle^3–5,7^. These pooled peptides when given with the TLR4 agonist adjuvant, GLA-SE (created by The Infectious Diseases Research Institute, IDRI) are able to produce protective memory CD8^+^ T lymphocytes that reduce the amounts of *T. gondii* in specific HLA transgenic mice. Therefore, we utilized GLA-SE as one of our adjuvants^8–10^. GLA-SE has an excellent pre-clinical track record and is in development through human clinical trials including for malignancies, viral, protozoan, and bacterial infections. Our previous work has demonstrated a superior effect of GLA-SE compared to ALUM^6,11^, when added to peptides eliciting cell mediated responses (CD8^+^ and CD4^+^ T lymphocytes) that protect against *Toxoplasma*. Our hypothesis is to create a safe and potent vaccine based on our preliminary immunosense and Self Assembling Protein Nanoparticle (SAPN) data, both with *Toxoplasma* infections and malaria^11–13^. We then embarked on engineering the peptides into SAPNs. These proteins serve as vaccine core platforms and have the ability to present immunogenic pathogen fragments to the host’s immune system (patent US8575110 B2^13–15^). These include CD8^+^, CD4^+^ T-, and B cells to promote strong cellular and humoral responses^12,16,17^. Because of their size and shape, they reach and are processed in follicular dendritic cells. They are flexible in design, and easy to produce rapidly. In addition, they do not present the risks of live attenuated vaccine strains. In our recent work (Prototype 1, Fig. 1a), we have engineered a SAPN that contains the *T. gondii* B07 binding epitope of the dense granule protein GRA7_20–28_ (LPQFATAAT) and an universal CD4^+^ T cell eliciting epitope (PADRE)^12^. Immunization of HLA-B*07:02 mice with these SAPNs, induced strong CD8^+^ T cell-dependent protective immunity against Type I and Type II *T. gondii* parasites, although protection is not complete. These findings highlight the development of a safe and effective T cell epitope-based toxoplasmosis vaccine. Furthermore, we developed a novel type of SAPN, called Prototype 2, that contains five HLA-A*11:01-restricted CD8^+^ T cell epitopes (Fig. 1a). This Prototype 2 incorporates the TLR5 agonist flagellin as a a scaffold and as an immunopotentiator to make a self-adjuvanting SAPN^11^ (patent application EP14150600^18^). This multiepitope construct has been shown to induce IFN-γ and protect against type II parasites in HLA-A*11:01 mice, demonstrating the ability of the SAPNs to improve vaccine potency of CD8-based immunization approaches^11^.

Here we present the designs and immunological profiling of a novel SAPN containing five CD8^+^ T cell eliciting HLA-A*11:01 binding protective epitopes, one CD8^+^ HLA-B*07:02, four CD8^+^ HLA- A*02:01, a pan-allelic CD4 epitope, and a MIC1 B cell epitope to yield the SAPN prototype called “*ToxAll*” (prototype 3, Fig. 1a). In addition to the introduction of several epitopes from three HLA supermotifs, another new aspect of our recent SAPN presented herein is the introduction of MIC1 as a B cell epitope (Fig. 1b). MIC1 was chosen because of it’s involvement in the interactions of the parasite with its host cell receptor during the early stages of host cell attachment, invasion, virulence and pathogenicity^19^. Hence, our study of *ToxAll* demonstrated the ability to elicit antibodies against the B cell epitope MIC1 in a humoral immune response and at the same time it delivered the specific HLA supermotif -restricted CD8^+^ and CD4^+^ epitopes to elicit a cellular immune response.

## Results

### Generation of SAPN nanoparticles

**Figure 2:**
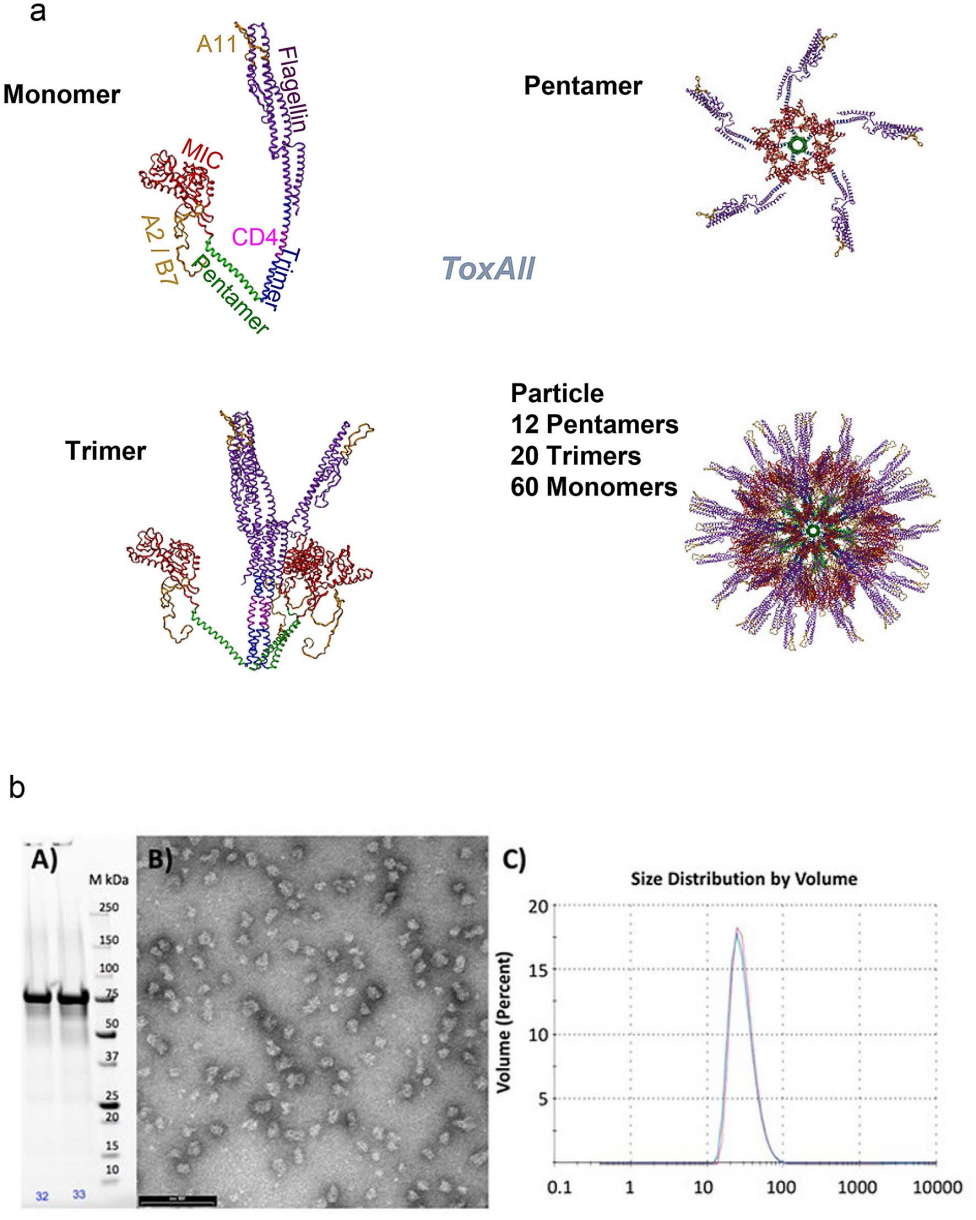
(**a**) Computer Model of *ToxAll*. The core particle is composed of the pentameric and trimeric coiled coils. They are shown in green and blue, respectively. Attached to the trimeric coiled coil is the TLR5 agonist flagellin (purple) while the B cell epitope MIC1 (red) is attached to the pentameric coiled coil. The HLA-A*11:01 CD8^+^ epitopes (gold) are engineered into flagellin, the CD4^+^ epitope is engineered into the trimeric coiled coil, the HLA-A*02:01 and HLA-B*07:01 CD8^+^ epitopes are attached to the N-terminal end of the protein chain. While the B-cell epitope MIC1 appears to be hidden in the assembled particle, the structures of the pentamer and trimer reveal that there is ample void space that allows antibodies to bind to MIC1 in the assembled structure. (**b**) Biochemical and Biophysical Analysis. Part (A) SDS-PAGE of the purified construct. Part (B) Transmission electron microscopy of *ToxAll* showing relatively uniform and non-aggregating nanoparticles. The bar corresponds to 100 nm. Part (C) DLS size distribution analysis of *ToxAll* showing an average nanoparticle size of ~ 40 nm.

Herein, we develop tools to improve our vaccine platform using a structure-based “plug and play” approach to the SAPN. We incorporated five CD8^+^ HLA-A*11:01 protective epitopes, one CD8^+^ HLA-B*07:02, four CD8^+^ HLA-A*02:01, a pan-allelic CD4^+^ epitope and a MIC1 B-cell epitope to yield the SAPN prototype called “*ToxAll*”. Figure 1b represents the sequence of different epitopes separated by proteasomal cleavage sites. We used the properties of trimeric and pentameric coiled coils sequences to plug and assemble the protective epitopes into a particle (Fig. 2a). HLA-A*11:01 CD8^+^ epitopes (gold) are engineered into flagellin. The CD4^+^ T cell eliciting epitope is engineered into the trimeric coiled coil. HLA-*A02:01, HLA-B*07:02, and CD4^+^ T cell eliciting epitopes are attached to the N-terminal end of the protein chain, while B-cell eliciting epitope, MIC1 appears to be hidden in the assembled particle. In a first step of our design, we attached the MIC1 protein to the N-terminal end of the protein chain of the SAPN (Fig. 2a). This MIC1 protein is presented on the tip of the pentameric coiled coil and displayed in a repetitive manner on the surface of the SAPN. The surface of the MIC1 protein that interacts with the cell receptor was exposed on the surface of the nanoparticles, thus inducing production of antibodies against the interaction site of MIC1. In a second step, we combined MIC1 SAPN with the prototype that carries the five HLA-A*11:01 epitopes, one HLA-B*07:02 and four HLA-A*02:01 T*. gondii* CD8^+^ T cell eliciting epitopes, the CD4^+^ T cell eliciting epitope PADRE intercalated into the flagellin portion of the design (Fig. 2a).

The *ToxAll* construct was expressed in *E. coli*. It was purified and folded to form nanoparticles as described in the “Materials and methods”. Figure 2b shows the molecular size of the protein ~ 75 kDa in Sodium dodecyl sulfate polyacrylamide gel electrophoresis (SDS-PAGE) with a uniform and non aggregate distribution of particles ~ 40 nm in diameter.

### SAPN that contains three HLA-A*02:01, one HLA-B*07:02, five HLA-A*11:01 CD8^+^ T cell eliciting epitopes, and the pan-allelic CD4^+^ T cell eliciting epitope PADRE, confers protection against *Toxoplasma*

**Figure 3:**
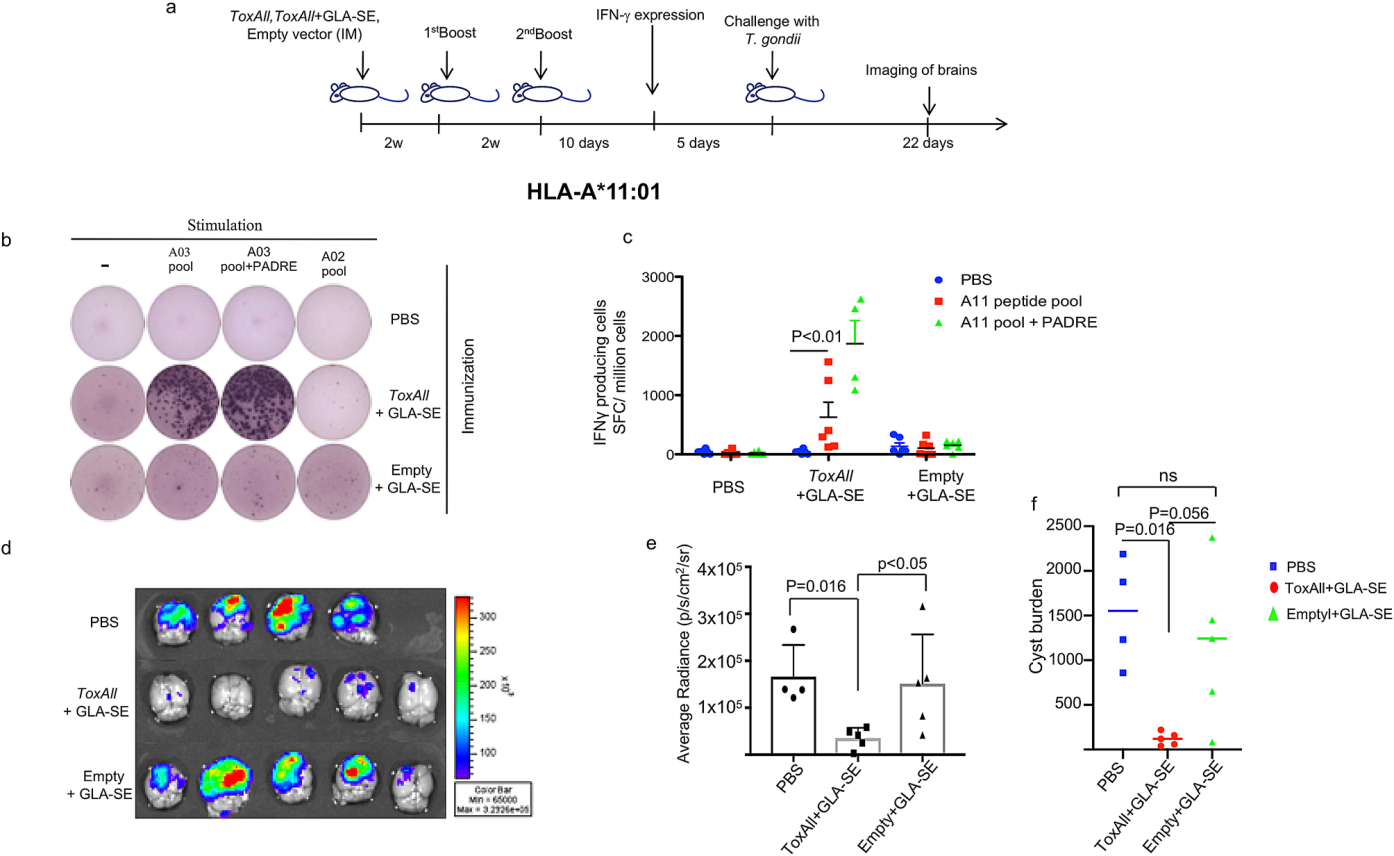
(**a**) Experimental design of Immunization of HLA-A*11:01 (and other) transgenic mice. (**b**, **c**) IFN-γ- producing T cells were measured by ELISpot assay. Mouse splenocytes were stimulated with pooled HLA-A*11:01 restricted CD8^+^ T cell epitopes (A03; HLA-A03 supertype allele of HLA-A*11:01) with or without PADRE. In some experiments as in the photograph of the well in ***b*** HLA-A*02:01 restricted CD8^+^ T cell peptides were used. There were 2 mice of the 6 in the *Tox-All* GLA-SE group that had splenocytes that had some response to these HLA-A*02:01 restricted peptides in contrast to the well shown in (**b**) (data not shown in photograph or graph of ELISpot data). There were 3 wells of splenocytes for each mouse. A mean value was determined for each mouse. (**b**,**c**) Present the data of the IFN-γ spot formation from 2 separate experiments combined. The numbers of mice were as shown in the figure with a total n = 4–6 mice per each group of controls or peptides utilized in vitro. Each symbol represents IFN-γ secretion for one mouse. The horizontal line represents the mean of these IFN-γ spot formation determinations**.** (**d**,**e**) Luciferase expression was reduced in the brains of HLA-A*11:01 mice that had been immunized with *ToxAll* plus GLA-SE. This was measured after challenge with *T. gondii* ME49-Fluc (Type II) expressing luciferase. Mice (n = 5 per experiment) were immunized, and challenged intraperitoneally 14 days after the last boost, with 2000 Type II (Me49-Fluc) parasites. Imaging was performed with the in vivo IVIS imaging system ( Xenogen, Alameda, CA) with surviving mice. The experiment was performed twice beginning with 5 mice per group. The numbers of mice that survived to the 21^st^ day when parasite burden as luminescence or (**f**) cyst number in brain were determined. (**f**) enumeration of cysts in mouse brain. Brains from these mice were collected and resuspended in saline and tissue cysts were counted using an optical microscope.

**Figure 4:**
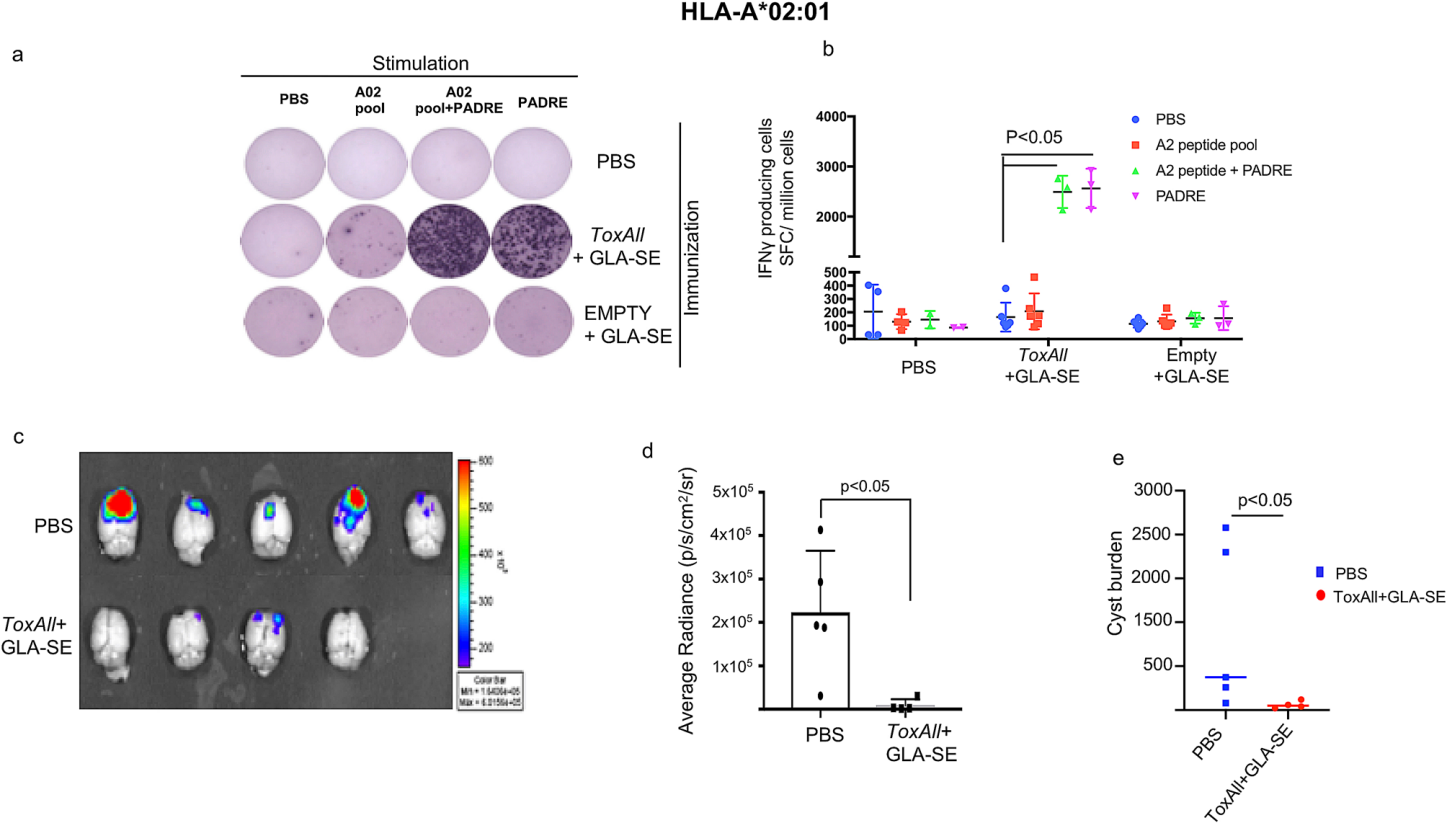
Protective immunity against toxoplasmosis induced by 4 pooled HLA-A*02:01 restricted CD8^+^ epitopes in HLA-A*02:01 mice. (**a**,**b**) IFN-γ producing T cells were measured by ELISpot assay,in the same manner as described in the legend of Fig. 3. Splenocytes from *ToxAll* plus GLASE and EMPTY SAPN plus GLA-SE immunized mice were stimulated with a pool of 4 HLA-A*02:01 restricted CD8^+^ T cell peptides in the presence or absence of PADRE, or PADRE alone. PBS group was used as a control. There was a trend toward a small response of splenocytes to HLA-A*02.01 restricted CD8 + T cell epitopes compared to PBS but it did not achieve statistical significance. The response in both groups with PADRE was significant. (**c**,**d**) *T. gondii* luciferase expression in brains *of* HLA-A*02:01 immunized mice *with* *ToxAll* plus GLA-SE was significantly reduced compared to group mice control (PBS). (**e**) Brains show the reduction of number of cysts in HLA-A*02:01 immunized mice compared to the control group.

**Figure 5:**
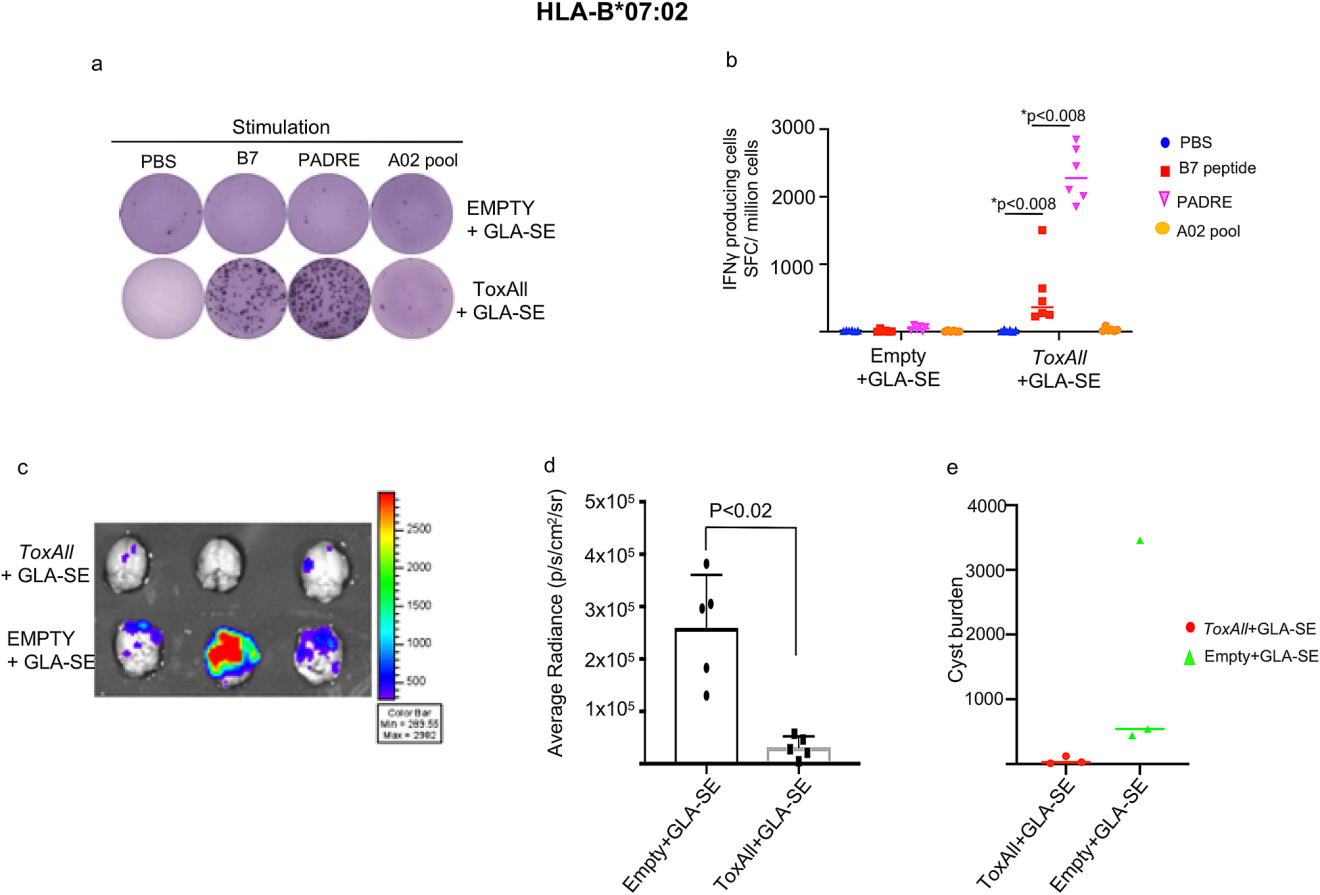
*ToxAll* elicits B07 epitope (GRA720–28, LPQFATAAT) specific immune response in HLA-B*07:02 mice*.* (**a**,**b**) show IFN-γ -producing T cells from splenocytes of HLA-B*07:02 mice immunized with *ToxAll* plus GLA-SE compared to Empty plus GLA-SE adjuvant. Data show the specificity of splenocytes from HLA-B*07:02 immunized mice with adjuvanted *ToxAll* to B07 epitope and not to the pool of HLA-A*02:01 restricted CD8^+^ peptides. The absence of any cross reactivity of HLA haplotypes demonstrates the specificity of that peptide HLA interaction and absence of any contribution of the heterologous combination. (**c**,**d**) Luciferase expression from brains of HLA-B*07:02 mice was significantly reduced in *ToxAll* immunized mice compared to control Empty-SAPN. (**d**) This reduction in luminescence for the *ToxAll* SAPN immunized transgenic mice correlates well with the reduction of number of cysts per brain counted shown in **e** (*p* = 1.0 because of one outlier in the control group with a much higher number of cysts but the trend show substantial reduction in cyst numbers for all mice by immunization with the *ToxAll* SAPN administered with GLA-SE).

The mice were immunized with either *ToxAll* with GLA-SE, Empty-SAPN with GLA-SE, or saline following the design presented in Fig. 3a. Immunogenicity was compared using magnitude of IFN-γ secretion from mouse splenocytes of HLA-A*11:01, HLA-A*02:01, and HLA-B*07:02. Figures 3, 4 and 5 show ELISpot experiment with IFN-γ spot formation from splenocytes, which were tested using a pool of HLA-A*11:01 or HLA-A*02:01 or HLA-B*07:02 restricted CD8^+^ T cell epitopes with or without PADRE and some mismatched HLA Class I restricted peptides as controls. Our data show that *ToxAll* elicits CD8^+^ T cells that respond to HLA-A*02:01, HLA-A*11:01, and HLA-B*07:02 binding constituent peptides and can present such antigens in a MHC restricted manner and CD4^+^ T cells that respond to PADRE. To test the properties of this SAPN in vivo, mice were challenged two weeks after the last immunization with *ToxAll* using 2000 Me49-Fluc, type II strain, *T. gondii*. Twenty-one days after the challenge, brains from these mice were removed and imaged using a Xenogen in vivo imaging system. As shown in Figs. 3d, 4c, and 5c, numbers of luciferase expressing parasites in brains of HLA-A*11:01, HLA-A*02:01, and HLA-B*07:02 transgenic mice immunized with *ToxAll* + GLA-SE were less (*p* < 0.05) in mice that received *Empty-SAPN* or PBS as controls. These correlate with the reduction of number of cysts per brain (Figs. 3f, 4e, and 5e). For experiments with two groups, between-group comparisons were made using the nonparametric Mann–Whitney test. For experiments with three or more groups, data were initially compared by the nonparametric Kruskal–Wallis test. If results from that were statistically significant, then relevant pairwise comparisons were made using GraphPad Prism 7 software (GraphPad Software, San Diego, CA). Results are expressed as the mean ± SD and considered statistically significant at *p* < 0.05.

### *ToxAll* induces humoral responses

**Figure 6:**
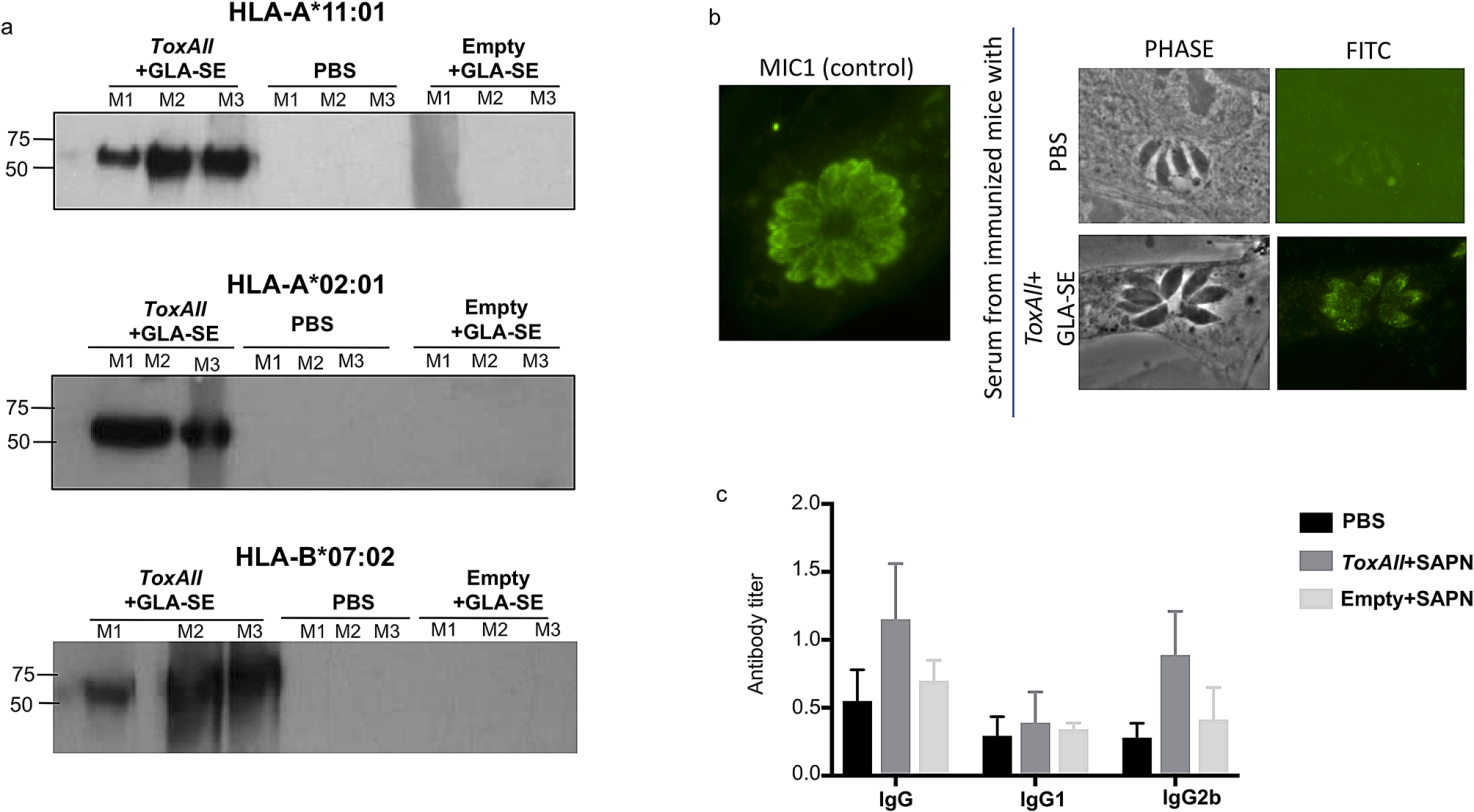
Antibody production. (**a**) Western blot analysis using protein extracts from ME49 strains with three sera from immunized HLA-A*11:01, HLA-A*02:01 mice, and HLA-B*07:02 that recognize MIC1 proteins. (**b**) Immuno Fluorescence Assay (IFA) using sera containing MIC1 from immunized transgenic mice in infected fibroblasts right panel. In comparison to MIC1 antibodies (from a MIC1 complete sequence used as a control), our data from the SAPN immunized mice with the truncated MIC sequence, do not show a staining at the surface of the parasite. This is consistent with the size of MIC1 plugged in *ToxAll*. (**c**) Detection of the levels of *T. gondii* specific antibodies and their subclass IgG1 and IgG2b. Serum samples were collected 10 days after the last immunization and antibodies level were tested by ELISA. Humoral responses were assessed by reading the mean of the optical density at 450 nm (OD_450_) ± SD values.

To determine whether *ToxAll* + GLA-SE elicits humoral responses by production of MIC1 antibodies, western blot analysis with sera, from immunized HLA-A*11:01, HLA-A*02:01, and HLA-B*07:02 transgenic mice was performed using total soluble antigen (STAg) fraction *T. gondii* from ME49 strains. As shown in Fig. 6a, the sera of the 3 immunized transgenic mice reacted only with the 60–70 kDa bands of STAg. The identity of these proteins were identified as MIC1 proteins by mass spectrometry analysis (data not shown). We performed also Immuno Fluorescence Assay (IFA) using sera from MIC1 immunized transgenic mice with infected fibroblasts and as a control, an anti MIC1 antibody. In comparison to the MIC1 antibodies (from a MIC1 complete sequence, used as a control), our data with the SAPN immune sera do not show a staining at the surface of the parasite, as described earlier^20^. This is consistent with the size of MIC1 plugged in *ToxAll* (Fig. 6b).

### Quantitation of levels of *T. gondii* specific antibodies and their subclass IgG1 and IgG2b

In order to determine the relative levels of IgG1 and IgG2a subclass reacting with *T. gondii* antigens, serum samples were collected from both immunized and non-immunized mice 10 days after the last booster immunization, and detected by ELISA. Compared with the control groups, there are higher levels of IgG and IgG2b antibodies in the sera from immunized mice (Fig. 6c). However, the level of IgG1 antibodies was not changed from immunized mice compared to the control group (Fig. 6c).

### Interaction of SAPN with lysosomal enzymes

**Figure 7:**
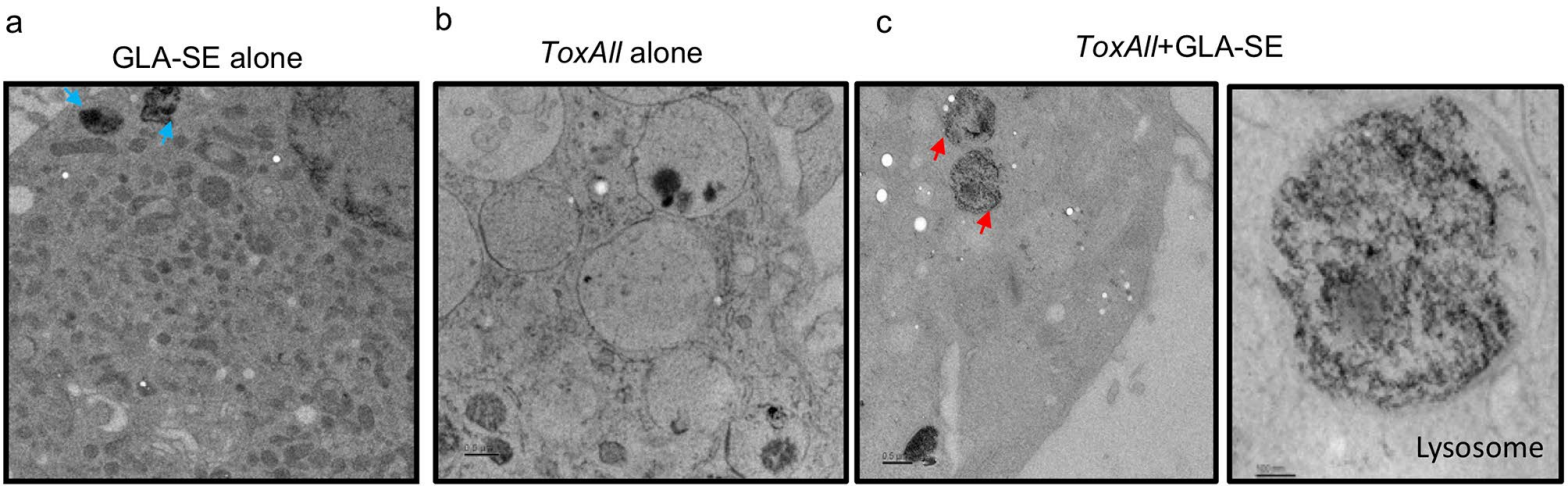
TEM images. After 24 h co-culture of bone marrow- derived dendritic cells (BMDDCs) from HLA-A*02:01 transgenic mice incubated with (**a**) GLA-SE alone, (**b**) *ToxAll* alone or (**c**) *ToxAll* with GLA-SE. GLA-SE present in particles mostly close to cell membranes with a size of ~ 100 nm (blue arrows). When incubated with *ToxAll* alone, lysosome of BMDDCs didn’t show any processing at 24 h. *ToxAll* plus GLA-SE interacts with lysosomal enzymes and shows a remarkably homogeneous distribution of particles in the lysosome. The red arrows indicate clusters of gold-encapsulated SAPN.

To understand the way in which the *ToxAll* processing and presentation is able to deliver T cell immune responses and protection described above, we analyzed in vitro bone marrow dendritic cells (BMDDCs) by electron microscopy. BMDDCs from HLA-A*02:01 transgenic mice were incubated for twenty-four hours with either *ToxAll* alone, *ToxAll* with GLA-SE or GLA-SE alone. Results indicate that while GLA-SE is processed at the plasma membrane (consistent with the interaction of the adjuvant to TLR4) (Fig. 7a), SAPNs plus GLA-SE interacts with lysosomal enzymes (Fig. 7c). There is a remarkable, homogeneous distribution and loading of antigenic peptides within the lysosome when SAPNs were added to the adjuvant and emulsion (Fig. 7c).

## Discussion

Herein, we engineered the multi-epitope toxoplasmosis nanovaccine *ToxAll* based on the SAPN concept. It contains five HLA-A*11:01 restricted CD8^+^ T cell eliciting epitopes, one HLA-B*07:02 restricted CD8^+^ T cell eliciting epitope, four HLA-A*02:01 restricted CD8^+^ T cell eliciting epitope, pan-allelic CD4^+^ T cell eliciting epitope for MHC II haplotypes, the TLR5 agonist flagellin, as well as the fully folded protein domain of MIC1 as a B cell epitope. This forms a SAPN that induced a combination of humoral immune stimulation, as well as cell mediated immune responses.

This construct is built based on Prototype 2 (Please see Fig. 1), which incorporates the TLR5 agonist flagellin as a component of the nanoparticle to make it a self-adjuvanting SAPN^11^. We found earlier that Prototype 2 elicits CD8^+^ T cells that respond to HLA-A*11:01 binding constituent peptides, and CD4^+^ T cells that respond to PADRE. This particular SAPN architecture contains TLR5 agonist flagellin as a scaffold and as an immunopotentiator (patent application EP14150600^18^) and these SAPN tritiate a robust innate immune response. This innate immune response is elicited through activation of the TLR5 pathway^11^. In the current construct, we combine HLA A02 and B07 supertypes from our earlier work^3–6^ to make a chimeric novel polypeptide with HLA-A*11:01 restricted CD8^+^ and a new B cell epitope microneme protein, MIC1. We also used our CD4^+^ T cell eliciting universal, but not parasite specific epitope, PADRE. We attached hydrophobic CD8^+^ T cell eliciting epitopes at the N-terminal end of the novel chimeric SAPN protein sequence so it would be located in the interior of the protein shell of the particle. Such location is preferred as it is predicted to avoid hydrophobic aggregation between particles in solution, similar to the hydrophobic core of correctly folded proteins. We had earlier unsuccessfully attempted to create linked HLA-A*02:01 restricted peptides as linear constructs as we did with HLA-A*11:01 peptides^6^ (data not shown).

Even though this final SAPN design looks rather complicated, herein we have successfully engineered a SAPN that combines MIC1, CD8^+^, CD4^+^ -T cell eliciting epitopes and flagellin in one single protein chain. This provides a framework that could be utilized to create other similar chimeric polypeptides including other immunogenic peptides and proteins which perhaps have potential to be multiplexed. This could broaden human population coverage still further and to protect against other organisms or diseases where similar immune responses are critical. We then could test them in these or other HLA transgenic mice where genetic restriction for those haplotypes would be operative.

All epitopes were flanked at the carboxy-terminus by N/KAAA spacers. These spacers lead to immunogenic processing that is optimal^6^. In separate earlier studies, we found that processing and presentation of five HLA-A*11:01 epitopes assembled with N/K alanine linkers in a single polypeptide (called LO poly-epitope) stimulated human cells occur with higher efficiency compared to individual peptides^6^, and conferred greater protection than the individual peptides did, or did a different combination of peptides with another linker (GPGPG)^6^. In the HLA-B*07:02, absence of any cross reactivity of HLA-A*02 haplotype peptide while HLA-B*07:02 is present demonstrates the specificity of that peptide HLA- B*07:02 interaction and absence of any contribution of the heterologous combination (Fig. 5). We had demonstrated earlier^6^ and again herein in Fig. 3 that the construct without its contents called “empty” herein had no difference from PBS in immunogenicity measured as stimulation in vitro by peptides.

The data show that PADRE clearly elicits a very robust response as an universal CD4^+^ T cell eliciting peptide known to stimulate CD4^+^ T cells strongly. Even with this strong response there is no parasite specific response, nor would one be expected, as PADRE is not present in *T. gondii*. PADRE is helpful in priming the initial response but there would be no recall in a natural infection. Thus, one component lacking in the final design is a *Toxoplasma*- epitope specific for HLA Class II alleles to elicit CD4^+^ T cells. We have embarked recently on the selection of these candidates from *T. gondii* that will be part of a final construct (data not shown).

These findings are important conceptually, especially because they show why this chimeric construct that we are building is in total more than the sum of its parts and this is proven in the work herein. This is significant for breadth of population coverage and for specificity of response matching peptide binding data and haplotype specific immune responses.

The results herein and in our earlier work have shown some variations in responsiveness even to the same peptides in terms of robustness at different times. In Figs. 3, 4, and 5, the scale of the vertical axis that shows very robust response to PADRE, makes these differences appear more pronounced. Although the response to PADRE is very robust and is important for stimulating IL2 production, which stimulates CD8^+^ T cells during the prime and boost immunizations, PADRE is not a *T. gondii* derived peptide. Since PADRE is not homologous, it will not drive a memory recall response relevant to *T. gondii* infection.

Additionally, the data with PADRE are important in the proof of principle that an epitope that elicits CD4^+^ T cells can be presented in this SAPN vaccine and elicits a strong response. The responses to the epitopes that elicit CD8^+^ T cells were not always robust compared to PADRE. Nonetheless, the genetic restriction demonstrated is critical for this proof of principle study. We had noted earlier that the linkers and arrangement of the peptides in the HLA-A*11:01 construct made the response greater than the 5 peptides separately in a linear construct and we retained that arrangement^6^. We had been unable to make a linear construct that folded the HLA-A*02:01 bound peptides. We selected an arrangement that allowed those hydrophobic HLA-A*02:01 epitopes to be on the inside face of the SAPN. Even more important than the magnitude of response that can vary or be made stronger by additional copies or linkers^6^, peptides bound by each HLA haplotype do indeed elicit a response specifically for that haplotype. This is the proof that homologous peptide present in the SAPN elicits the appropriate HLA-restricted T cell response, although in this study the HLA-02 restricted response is modest, along with the MIC protein being able to elicit antibody.

MIC1 has a tandemly duplicated domain related to thrombospondin-1-like domain of thrombospondin that binds to or alters host cell receptors^21^. Homology of these MIC1 containing domains were found in *P. falciparum* circumsporozoite protein (PfCSP). When these domains of PfCSP were used in SAPNs they elicited antibodies and protected against *P. falciparum*, suggesting that MIC1 may be useful in protection against *T. gondii*. A SAPN vaccine has been created for malaria. This vaccine for this disease caused by the related apicomplexan plasmodial parasites, was created to immunize with PfCSP derived epitopes eliciting T and B cells^13,22^. Moreover, the crystal structure of MIC1 protein has been solved^23^. This crystal structure allows the design of specific vaccine components that will induce immune responses that interfere with/impair parasite interactions with host cells; i.e. antibody raised against the surface of MIC1 that interacts with the host receptor will impair parasite interactions dependent on MIC1 with host cells. We have placed MIC1 in our SAPN in order to make an antibody with potential to contribute to blocking the interaction of the parasite with the host^24^. MIC proteins recently were used as recombinant vaccines that conferred some protection^25^. Although *ToxAll* induces humoral responses, sera containing MIC1 from immunized HLA-A*11:01, HLA-A*02:01, and HLA-B*07:02 transgenic mice do not block parasite invasion into fibroblasts (data not shown). MIC1, MIC4 and MIC6 complex has been shown to be critical in invasion^26^. Thus an antibody to these MIC proteins might contribute to blocking invasion of the host cells as did antibodies critical to invasion such SAG1^27^, and AMA-RON^28^.

Interestingly, *ToxAll* plus GLA-SE interacts with lysosomal enzymes (Fig. 7c). There is a remarkable, homogeneous distribution and accumulation of antigenic peptides within the lysozome when SAPNs were added to the adjuvant and emulsion. The results for this localization and pattern are consistent with a similar study performed earlier with *Plasmodium falciparum*^29^. It will be important to study the specific kinetics of the processing of *ToxAll* through BMDDCs. Specifically, and in addition with TEM, confocal microscopy could be examined to support the cleavage of the peptides within the *ToxAll* SAPN processing during the twenty-four hours. This would contrast efficiency of processing of *ToxAll* + GLA-SE with *ToxAll* alone in different organelles (e.g. endoplasmic reticulum, endosome/lysosome).

Additionally, our SAPN contains flagellin. It is expected that our new SAPN design, with flagellin in its scaffold will retain TLR5 activity. In ongoing separate work using another immunization platform^30^, we included the sequence for *ToxAll* in a RNA replicon. In this system we found that flagellin encoded in this replicon construct does stimulate TLR5.

Herein, challenges with Me49 type II parasites in HLA-A*11:01, HLA-A*02:01, and HLA-B*07:02 mice demonstrated reduction (87%) from a mean of 1550 to 200 cysts per brain. This correlates with our previous studies that showed also reduction of cysts in brains of mice^6,11,12^, but not as robustly as our *ToxAll*. However, none of these vaccine regimens provided complete protection. More peptides will be needed for sufficient population coverage, and possibly multiple copies with these peptides, or to elicit a still more robust response. Nonetheless, these experiments demonstrate that the overall structure can present peptides and proteins to the appropriate compartments to elicit a protective immune response. These critical concepts are illustrated by the data in Figs. 3, 4, 5 and 6 and shape the approaches in our next steps in development of a vaccine.

## Conclusion

To our knowledge, the *ToxAll* presented here represents the first SAPN for the delivery of CD4^+^, CD8^+^ T cell eliciting and B cell eliciting epitopes with testing in different HLA transgenic mice.

First, we investigated the folding and the size of the SAPNs by DLS size distribution and electron microscopy analysis. We assessed the functionality of the SAPNs by mixing them with GLA-SE adjuvant. GLA-SE has been recently used extensively in human clinical trials. GLA-SE is one of the oil-in-water (o/w) emulsions developed at IDRI. Emulsion droplets are ~ 100 nm in diameter and are stable for years. This IDRI emulsion formulation is similar to formulations already approved in Europe for influenza vaccines, MF59 and AS03, but in addition contains TLR4 ligand GLA. Emulsions like these effectively and safely induce immune responses to influenza antigens, including enabling dose sparing^31–33^. The mice were immunized with either *ToxAll* with the adjuvant GLA-SE, Empty-SAPN with GLA-SE, GLA-SE only, or PBS. First, we used PBS as a control in these studies in HLA-A*11:01 mice. Immunogenicity was compared using the magnitude of IFN-γ production by mouse splenocytes ex vivo. We showed that IFN-γ secretion is high in mice immunized with *ToxAll* plus GLA-SE stimulated by either a pool of peptides or PADRE. To evaluate whether immunization with *ToxAll* confers any protection against the Type II strain of *T. gondii* (Me49-Fluc), brains of immunized mice were imaged using a Xenogen in vivo imaging system. We showed that immunized HLA-A*11:01 transgenic mice that received *ToxAll* had significantly reduced parasite numbers in their brains compared to mice that received the control *Empty-SAPN*, adjuvant alone, or PBS. This is consistent with the reduction of cysts in the mice immunized with *ToxAll* (*p* < 0.05). Thus, our multi-epitope *ToxAll* elicited protection quantitated as reduction of parasite cyst burden and enhanced survival when administered with GLA-SE laying the foundation for creating a novel type of nanovaccine, to reduce or eliminate initial *T. gondii* parasite burden.

Understanding the mechanisms whereby CD4^+^ and CD8^+^ T cell, and B cell restricted epitopes are associated with protection of HLA-A*11:01, HLA-A*02:01, and HLA-B*07:02 mice against toxoplasmosis, underline the role of the *ToxAll* scaffold in the rational design of a T cell- epitope based vaccine strategy. This represents a novel approach to generate host neutralizing antibodies against different parasites and in other diseases.

## Materials and methods

### Cloning *ToxAll* gene

NdeI/EcoRI restriction sites were used to clone the polypeptide in pET23b vector (Novagen). *ToxAll* has the sequence shown in Fig. 1b as follows: A his-tag sequence (black); 4 HLA-A*02:01 restricted CD8^+^ epitopes FMGVLVNSL (GRA629-37), ITMGSLFFV (SRS52A12-20), FMIVSISLV (SAG2X351-360), GLAAAVVAV(SPA82-90) (gold); one HLA-B*07:02, LPQFATAAT (GRA7_20-28_) (gold); the B cell epitope microneme protein 1 (MIC1) of *T. gondii* p89 (red); the pentameric coiled coil (green); within the trimeric coiled coil (blue), pan-allelic CD4^+^ epitope string (magenta); five HLA-A*11:01 restricted CD8^+^ T cell epitopes represented in (gold) AVVSLLRLLK(GRA5_89-98_), AMLTAFFLR(GRA6_164-172_), KSFKDILPK(SAG1_224-232_), STFWPCLLR(SAG2C_13-21_), SSAYVFSVK(SRS52A_250-258_). These epitopes were placed into the domain D2 and D3 of the flagellin sequence((purple) of *Salmonella enterica*. This flagellin has the structure that has pdb-code 3V47^34^. This is from RCSB protein data bank.

A control construct, designed as empty vector contains all the peptide sequences except HLA restricted CD8^+^, CD4^+^ eliciting and MIC1 epitopes.

### Protein expression, purification, refolding, and analysis of the nanoparticle polypeptide

The construct called *ToxAll* was expressed in *E. coli* BL21 (DE3) cells as described in our previous work 3,5. Expression clones were grown at 37 °C in Luria broth media with ampicillin. Protein expression was induced by the Isopropyl β-D-1-thiogalacto-pyranoside (IPTG) to an OD_600_ of ~ 0.8. After induction at ~ 4 h, cells were removed from 37 °C and centrifuged at 4000×*g*. Cell pellet was stored at − 80 °C. The cell pellet was thawed on ice, suspended in a lysis buffer containing 9 M urea, 10 mM Tris pH 8, 100 mM NaH_2_PO_4_, 20 mM imidazole, and 0.2 mM of the reducing agent Tris-2-carboxyethyl phosphine (TCEP), sonicated, and then centrifuged at 30,500×*g* for 45 min to clear the lysate. The His-tagged Recombinant protein was purified by using nickel-affinity chromatography followed by Q-Sepharose. The eluate *ToxAll* protein was first rebuffered in denaturant conditions to the following conditions: 8 M urea, pH 8.5, 20 mM Tris, 50 mM NaCl, 5% Glycerol, 5 mM TCEP, and then refolded in buffer composed of pH 7.5, 20 mM Tris, 50 mM NaCl, and 5% Glycerol. After refolding, the final protein concentration was 0.05 mg/mL using Bradford method. The shape and size of the SAPNs was analyzed using Transmission Electron Microscopy (TEM) and Dynamic Light Scattering (DLS).

### Immunizations of mice and quantitation of parasite burden in brains of mice as numbers of cysts after challenge with type II parasites

The mice used in this study were female HLA-A*11:01, HLA-A*02:01 and HLA-B*07:02 transgenic mice. HLA-A*1101/K^b^, HLA-A*0201/K^b^ and HLA-B*0702/K^b^ were produced originally at Pharmexa-Epimmune (San Diego, CA) on a C57BL/6 (for HLA-A*11:01 and HLA-B*02:01) and C57BL/6 × Balb/C (for HLA-B*07:02) background, embryo-rederived at JAX laboratories and now are bred at the University of Chicago and JAX laboratories. To evaluate the immunogenicity of the SAPNs, transgenic mice were inoculated intramuscularly (i.m.) with 20 μg SAPNs emulsified in 5 μg of GLA-SE three times at two week intervals. For challenge studies, 5 mice per group were immunized, challenged intraperitoneally 14 days after the last boost with 2000 Type II (Me49-Fluc) parasites. They were imaged 21 days after challenge. This imaging was performed using an in vivo imaging system. This system is made by IVIS (Xenogen, Alameda, CA). The experiment was performed twice with 5 mice per group. To proceed, Mice were injected retro-orbitally with luciferin. Specifically, 200 μl of D-luciferin was injected. Mice were anesthetized in an O2-rich induction chamber. Anesthesia was 2% isoflurane. They were imaged 12 min later. Mice brains were analyzed ex-vivo for imaging. Assessment of photonic emissions was performed using software called Living image® 2.20.1 produced by Xenogen. This experiment was performed twice with 5 mice in each group. Mice were euthanized at 21 days after challenge. Brains were collected and resuspended in 1 ml of saline (0.85% NaCl) and tissue cysts were counted in 50 μl of homogenate using optical microscope. The obtained number was multiplied by 20 to obtain the tissue cysts per mouse brain. Dolichos biflorus agglutinin was used in parallel for the confirmation of cysts.

### ELISpot assay on murine splenocytes

Harvested spleens were pressed through a screen. This was a 70 μm screen . This created a single-cell suspension. Then, this suspension was depleted of erythrocytes by using AKC lysis buffer. The AKC buffer contained 10 mM KHCO3, 160 mM NH4Cl, and 100 mM EDTA. Following washing splenocytes twice using Hank’s Balanced Salt Solution (HBSS) splenocytes were resuspended in complete RPMI medium. RPMI-1640 was supplemented with 2 mM L-GlutaMax. We then performed an IFN-γ enzyme linked immunospot (ELISpot) assay. For this assayα we used α-mouse IFN-γ mAb (AN18). We also used biotinylated α-mouse IFN-γmAb (R4–6A2)^4,6,11^. The reagents and antibodies that we used for the ELISpot assay were from Mabtech, Inc (Cincinnati, OH). 2.5–5 × 10^5^ splenocytes were placed per well . We used at least three replicate wells for each of the conditions. Our results are expressed as the numbers of SFCs (spot forming cells) per 10^6^ mouse splenocytes. There were 3 mice per group, with 3 determinations per mouse with each experiment performed two separate times (n = 6 in total).

### Measurement of humoral IgG subclass responses

Ten days after the second immunization, we measured the isotype and antibody to MIC1. Levels of serum antigen specific to IgG1 and IgG2b antibodies were determined by ELISA, as described earlier^35^. Briefly, 50 µg of tachyzoite Antigen Lysate (TLA) in 50 mM carbonate buffer (pH 9.6) was adsorbed overnight onto 96 well plates (Corning Incorporated, Corning, NY). The plates were blocked with a blocking buffer composed of 1% of milk in saline and 0.05% Tween 20 (PBST) and incubated with the mouse serum (diluted with PBS 1:25) for 2 h at 37 °C. Then, wells were washed three times with PBST and individual anti mouse IgG-IgG1 and IgG2b conjugated to horseradish peroxidase (HRP) (Sigma-Aldrich) sera was added for 1 h at 37 °C. 3,3′, 5, 5′-tetramethylbenzidine (TMB) from (Sigma-Aldrich) was used to detect the peroxidase activity. After blocking the experiment with 2 M H_2_SO_4_, the optical density at 450 nM (OD = 450 nm) was recorded using a microplate fluorimeter.

Immunoblot analysis of MIC1 reacting with the serum from immunized mice was performed using tachyzoite Antigen Lysate (TLA) in 3 mice per group. This experiment was performed twice. In brief, 50 µg of TLA were suspended in sodium dodecyl 10% sulfate polyacrylamide gel electrophoresis (SDS-PAGE), electro-blotted to a nitrocellulose membrane and probed with serum from immunized mice diluted 1/50 in PBS-T containing 1% BSA, for 1 h, at 24 °C. Reacting antibodies were detected using 1/5000 peroxidase-conjugated goat anti-mouse IgG (Sigma), for 1 h, at 24 °C and reactions were visualized with DAB substrate kit (Pierce). For IFA, we used sera from mice and anti MIC1 antibody kindly provided by Dr. M. Lebrun, France.

### Electron microscopy

To better understand the properties of *ToxAll* for the delivery of immunogenic epitopes, we analyzed by electron microscopy the processing and presentation of *ToxAll* in bone marrow derived dendritic cells (BMDDCs). BMDDCs were cultured overnight with 20 µg of SAPN in the presence or absence of 5 μg of GLA-SE. The cells were harvested and spun at 300 × g for 10 min. The cell pellet was placed into planchettes (Ted Pella), cryo-fixed using a High-Pressure Freezing (HPF) Machine, and placed immediately into cryo-tubes containing a frozen cocktail of 0.1% uranyl acetate and 0.25% glutaraldehyde in anhydrous acetone. Samples were frozen, washed with acetone, infiltrated with Lowicryl resin, and mounted on 300 mesh copper grids (EMS). Images were collected on an electron microscope operated at 120 kV (Tecnai Spirit; FEI) with a 2 K ultra scan digital camera (Gatan).

### Statistical analysis

For each assay, groups include untreated or mock treated controls. For experiments with two groups, between-group comparisons were made using the nonparametric Mann–Whitney test. For experiments with three or more groups, data were initially compared by the nonparametric Kruskal–Wallis test. If results from that were statistically significant, then relevant pairwise comparisons were made using GraphPad Prism 7 software (GraphPad Software, San Diego, CA). Results are expressed as the mean ± SD and considered statistically significant at *p* < 0.05.

### Ethics approval

All methods were carried out in accordance with relevant guidelines and regulations. Animal experiments were performed with the review and approval of the Institutional Care and Committee at the University of Chicago (AICUC# 71734).

## Data Availability

All data will be shared.
